# DEGRO practical guideline for partial-breast irradiation

**DOI:** 10.1007/s00066-020-01613-z

**Published:** 2020-04-29

**Authors:** V. Strnad, D. Krug, F. Sedlmayer, M. D. Piroth, W. Budach, R. Baumann, P. Feyer, M. N. Duma, W. Haase, W. Harms, T. Hehr, R. Fietkau, J. Dunst, R. Sauer, Breast Cancer Expert Panel of the German Society of Radiation Oncology (DEGRO)

**Affiliations:** 1 University Hospital Erlangen, Erlangen, Germany; 2 University Hospital Schleswig-Holstein, Kiel, Germany; 3 Paracelsus Medical University Hospital Salzburg, Salzburg, Austria; 4 Helios University Hospital Wuppertal, Witten/Herdecke University, Wuppertal, Germany; 5 Heinrich-Heine-University Hospital Düsseldorf, Düsseldorf, Germany; 6 St. Marien-Krankenhaus Siegen, Siegen, Germany; 7 Vivantes Hospital Neukoelln, Berlin, Germany; 8 University Hospital, Jena, Germany; 9 St.-Vincentius-Hospital Karlsruhe, Karlsruhe, Germany; 10 St. Claraspital Basel, Basel, Switzerland; 11 Marienhospital Stuttgart, Stuttgart, Germany

**Keywords:** Breast cancer, Partial breast irradiation, Guideline, APBI, Early breast cancer

## Abstract

**Purpose:**

This consensus statement from the Breast Cancer Working Group of the German Society for Radiation Oncology (DEGRO) aims to define practical guidelines for accelerated partial-breast irradiation (APBI).

**Methods:**

Recent recommendations for relevant aspects of APBI were summarized and a panel of experts reviewed all the relevant literature. Panel members of the DEGRO experts participated in a series of conferences, supplemented their clinical experience, performed a literature review, and formulated recommendations for implementing APBI in clinical routine, focusing on patient selection, target definition, and treatment technique.

**Results:**

Appropriate patient selection, target definition for different APBI techniques, and basic rules for appropriate APBI techniques for clinical routine outside of clinical trials are described. Detailed recommendations for APBI in daily practice, including dose constraints, are given.

**Conclusion:**

Guidelines are mandatory to assure optimal results of APBI using different techniques.

## Introduction

To date, the gold standard for local treatment of patients aged 50 years or more with early breast cancer (pT1–2, pN0) and low-risk factors is breast-conserving surgery followed by postoperative whole-breast irradiation (WBI), typically without tumor-bed boost irradiation, which requires a treatment time of 3–6 weeks. Nowadays partial-breast irradiation (PBI) is also proposed for these patients; however, this strongly depends on the technique used [1–8]. PBI is a treatment approach able not only to shorten the course of radiation therapy (RT), but also to reduce the radiation exposure to the lung, the heart, the breasts, and the skin significantly, depending on the treatment technique [9–11]. Over the past 20 years, different modalities of PBI have been tested, mostly successfully, in a number of phase 2 and 3 clinical trials. The studied techniques are external beam radiation (photons, protons), single- and multicatheter brachytherapy, electronic brachytherapy, seed brachytherapy, non-invasive brachytherapy, and intraoperative radiation techniques (IORT) either with electrons or with 50-kV photons. Today, results from over 15,000 patients recruited within phase 3 trials testing partial-breast irradiation are available [1, 2, 5, 6, 12–19] and as a consequence, selected PBI techniques have been introduced into daily clinical routine. However, only small trials deal with PBI techniques using protons, brachytherapy (BT) using seeds, non-invasive BT, or numerous single-catheter devices. Thus, for the purpose of this guideline, we only analyzed techniques tested in randomized phase 3 trials.

## Methods

The authors evaluated the relevant literature, identified established and controversial topics via working conferences, circular emails, and meetings, and supplemented this information with their clinical experience to formulate the current guidelines. This report document was reviewed and approved by the full panel, and by the DEGRO Executive Committee. Currently available phase 3 trials are listed in Table 1.

**Table 1:** Summary of currently available Phase 3 PBI trials

Trial	Treatment		Patients (*n*)	Local recurrence (%)		Disease-free survival (%)		Overall survival (%)		Follow-up (median)
	WBI	PBI		WBI	PBI	WBI	PBI	WBI	PBI	
Barcelona [5]	48 Gy/24 fr. ± boost	10 × 3.75 Gy 3D-RT in 5 days	102	n.a.		n.a.		n.a.		5.0 years
Florence [16]	50 Gy/25 fr. + boost	5 × 6 Gy IMRT in 2 weeks	520	1.5 vs. 1.5 *p* = 0.86		n.a.		96.6 vs. 99.4 *p* = 0.057		5.0 years
IMPORT-LOW [2]	40 Gy/15 fr.	40 Gy/15 fr. IMRT	2018	1.1 vs. 0.5 *p* = 0.016*		n.a.		94 vs. 94 *p* = 0.69		6.02 years
RAPID [13, 18, 21]	50 Gy/25 fr. or 42.5 Gy/16 fr. + boost	10 × 3.85 Gy 3D-RT in 5 days	2135	2.8 vs. 3.0 n. s.		n.a.		n.a.		8.6 years
NSBAP B‑39 [12, 17]	50 Gy/25 fr. + boost	10 × 3.85 Gy 3D-RT or HDR siBT or BT in 5–10 days	4216	3.9 vs. 4.6 n.a.		79.7 vs. 78.1 *p* = 0.10		91.3 vs. 90.6 *p* = 0.35		10.2 years
Budapest [23]	50 Gy	BT: HDR 7 × 5.2 Gy in 4 days; or EB 50 Gy in 5 weeks	258	5.1 vs. 5.9 *p* = 0.77		85 vs. 84 n. s.		80 vs. 82 n. s.		10.2 years
GEC-ESTRO [1, 3, 4]	50 Gy/25 fr. + boost	HDR-BT 8 × 4 Gy or 7 × 4.3 Gy in 5 days; or PDR-BT 50 Gy in 5 days	1184	0.9 vs. 1.4 *p* = 0.42		94.4 vs. 95.0 *p* = 0.79		95.5 vs. 97.3 *p* = 0.11		6.6 years
ELIOT [15]	50 Gy/25 fr. + boost	IORT 1 × 21 Gy	1305	0.4 vs. 4.4 *p* < 0.0001		n.a.		96.9 vs. 98.8 *p* = 0.59		5.8 years
TARGIT [14]	50 Gy/25 fr. ± boost	IORT 1 × 20 Gy	3451	1.3 vs. 3.3 *p* = 0.042		n.a.		94.7 vs. 96.1 *p* = 0.099		29 months

*n*.*a.* not available/not reported, *n.* *s.* not significant, *fr.* fractions, *IMRT* intensity-modulated radiation therapy, *EB* electron beam, *WBI* whole breast irradiation, *PBI* partial-breast irradiation, *3D-RT* conformal external beam, *BT* multicatheter brachytherapy, *HDR* high-dose-rate, *PDR* pulse-dose-rate, *siBT* single device brachytherapy (MammoSite, Cytyc Corporation, Palo Alto, CA)

*p-value for non-inferiority

## Results

### External beam radiation therapy

The use of external beam radiation therapy (EBRT) for PBI appears to be very attractive, because this technique is broadly available worldwide. In two small randomized trials from Barcelona and Florence [5, 16] with 105 and 520 patients, respectively, patients were either treated with using intensity-modulated EBRT for PBI or using WBI. In general, similar efficacy (local recurrence rate, disease-free survival, and overall survival), toxicity, and cosmetic outcome were reported. However, the statistical power of both trials to prove non-inferiority of recurrence rates was inadequate.

Other trials—IMPORT LOW, RAPID, and NSBAP-B-39/RTOG 0413—studied sufficient numbers of patients.

IMPORT LOW [2] is a multicenter, randomized, controlled, phase 3, non-inferiority trial done in the UK. Patients were randomly assigned to receive 40 Gy in 15 fractions of whole-breast radiotherapy (control), 36 Gy in 15 fractions of whole-breast radiotherapy with a simultaneous integrated boost to 40 Gy to the tumor bed (reduced-dose group), or 40 Gy to the partial breast only (partial-breast group) also in 15 daily treatment fractions. For localization of the tumor bed, surgical clips were preferably used, but if this was not possible, ultrasound, MRI, or CT was used [20]. Field-in-field intensity-modulated radiotherapy was delivered using standard tangential beams that were simply reduced in length for the partial-breast group. The protocol specified forward-planned field-in-field IMRT delivered by standard medial and lateral tangential beams reduced in length but not in width. Non-target breast tissue medial or lateral to the planning target volume was thereby included in the high dose zone. Altogether, 2018 women were recruited. 674 patients were analyzed in the whole-breast radiotherapy (control) group, 673 in the reduced-dose group, and 669 in the partial-breast group. After a median follow-up 72.2 months, the cumulative 5‑year local relapse incidence was 1.1% in the control group, 0.2% in the reduced-dose group, and 0.5% in the partial-breast group; hence, a non-inferiority of PBI using 2.66 Gy in 15 fractions in 3 weeks was confirmed. Patient and clinical assessments recorded similar adverse effects after reduced-dose or partial-breast radiotherapy, including two patient domains achieving statistically significantly lower adverse effects (change in breast appearance [*p* = 0.007 for partial-breast] and breast harder or firmer [*p* = 0.002 for reduced-dose and *p* < 0.0001 for partial-breast]) compared with whole-breast radiotherapy. Thus, for PBI in 3 weeks, equivalent or fewer late normal tissue adverse effects were seen. The IMPORT LOW trial is the only phase 3 trial of partial-breast radiotherapy to use the same overall treatment time and radiation technique in the whole-breast and partial-breast radiotherapy groups. Because the same regimen is used, differences in treatment outcome can be attributed to differences in treatment volume [2].

In the RAPID trial [13, 18, 21] with altogether 2135 patients and a median follow-up of 8.6 years, patients aged >40 years with invasive or in situ breast cancer ≤3 cm were randomly assigned after breast-conserving surgery to 3D-CRT APBI or WBI. WBI was delivered daily to 42.5 Gy in 16 fractions or 50 Gy in 25 fractions using tangential fields. Additional boost irradiation of 10 Gy in four to five fractions after WBI was based on criteria such as young age or close margins. Patients allocated to APBI were treated with three to five noncoplanar conformal fields. The clinical target volume was the tumor bed on computed tomography, including the surgical clips plus a 1-cm margin inside breast tissue. The planning target volume was the clinical target volume plus a 1-cm margin. The dose-evaluation volume was the subvolume of the planning target volume inside breast tissue. The prescribed dose was 38.5 Gy in 10 fractions treated twice daily over 5 to 8 days [13, 21]. After a median follow-up of 36 months, adverse cosmesis at 3 years was increased among those treated with APBI compared with WBI, as assessed by trained nurses (29% vs. 17%; *p* < 0.001), by patients (26% vs. 18%; *p* < 0.0022), and by physicians reviewing digital photographs (35% vs. 17%; *p* < 0.001; [21]). The most recent analysis after a median follow-up of 8.6 years confirmed these findings: accelerated partial-breast irradiation (APBI) using three-dimensional conformal radiotherapy (3D-CRT) significantly increased grade 2 (28% APBI vs. 12% WBI) and grade 3 late radiation toxicity (4.5% APBI vs. 1% WBI, *p* < 0.001) and adverse cosmesis (31% vs. 15%; *p* = 0.001). Nonetheless, APBI vs. WBI showed similar oncological efficacy: local recurrence rate 2.3% vs. 1.7% after 5 years and 3.0% vs. 2.8% after 8 years. These data were presented at the San Antonio Breast Cancer Symposium 2018 [13] and published recently [18]. Thus, this trial confirmed non-inferiority of APBI using EBRT to WBI in preventing local recurrence, but because of increased late side effects and adverse cosmesis, the authors were unable to recommend the twice-a-day regime over 5 days for APBI using EBRT.

In the NSBAP-B-39/RTOG 0413 [12, 17] phase 3 trial, a total of 4216 patients with early breast cancer were randomized to whole-breast irradiation with 50 Gy (1.8–2.0 Gy/fraction) followed by an optional boost to ≥60 Gy or to partial-breast irradiation in 10 treatments given over 5 to 10 days (34 Gy in 3.4-Gy fractions using interstitial brachytherapy or Mammosite balloon catheter or 38.5 Gy in 3.85-Gy fractions using 3D conformal external beam). There were 24% of patients with pure DCIS and 10% of patients with positive lymph nodes among the recruited patients. Furthermore, in the WBI arm, 80% of patients received boost irradiation and in the PBI arm, the most frequently used technique was 3D conformal EBRT (71%) and the MammoSite (Cytyc Corporation, Palo Alto, CA) single-entry device (23.3%). Only 5.7% of patients received multicatheter brachytherapy as the PBI technique. After a median follow-up of 10.2 years, similar cumulative incidences of in-breast recurrences (4.6% APBI vs. 3.9% WBI), distant disease-free survival (96.7% APBI vs. 97.1% WBI, *p* = 0.1), and overall survival (90.6% APBI vs. 91.3% WBI, *p* = 0.35) were observed [12, 17]. In this trial, the intent-to-treat and as-treated analyses could not refute the hypothesis that PBI is inferior and cannot declare that WBI and PBI are equivalent in controlling local in-breast tumor recurrence. However, the absolute difference in the 10-year cumulative incidence of IBTR was only 0.7%. An important and notable finding was that upon analyzing only true early breast cancer patients using ASTRO criteria [22], the 10-year cumulative in-breast recurrence rate was significantly lower in both arms (2.7% APBI vs. 2.3% WBI). In addition, slightly different non-significant rates of grade 3 (9.6% APBI vs. 7.1% WBI) and grade 4–5 toxicity (0.5 APBI vs. 0.3% WBI) late side effects were reported.

Of note, the target volumes of external APBI seem to differ remarkably between IMPORT LOW and the American trials. In the IMPORT trial, target volumes were generously designed, also due to the fact that surgical clip demarcation of the tumor bed was not mandatory. Treatment was performed mostly by tangential field techniques, ending up rather in “half-breast” irradiations. In the RAPID as well as in the NSBAP-B-39/RTOG 0413 trial, despite similar PTV definitions, treatment delivery was more conformal by the use of several non-coplanar fields, thus encompassing less tissue. Unfortunately, none of the authors have so far provided absolute dimensions of treated volumes for the study patients.

### Brachytherapy

The use of multicatheter interstitial brachytherapy for APBI has been tested in two phase 3 trials so far.

Polgar et al. [23] randomized 258 patients with early-stage invasive breast cancer to receive either WBI or APBI with multicatheter HDR brachytherapy or with electron beam irradiation. After a median follow-up of 10.2 years, the 10-year rate of local recurrence was 5.9% and 5.1% in the APBI and WBI arms, respectively. The rate of excellent to good cosmetic results was 81% in the APBI, and 63% in the control group (*p* < 0.01). However, the statistical power of the trial regarding non-inferiority is limited due to the number of randomized patients.

In the Group Européen de Curiethérapie/European Society for Radiotherapy and Oncology (GEC-ESTRO) multicentric phase 3 trial [1, 3, 4], a total of 1184 patients were randomized to WBI or APBI using multicatheter brachytherapy. Patients were considered eligible for the trial if they were aged 40 years or older; had pTis or pT1–2a (lesions of ≤3 cm diameter), pN0/pN1mic, and M0 breast cancer (stage 0, I, and IIA); had undergone local excision of the breast tumor with microscopically clear resection margins of at least 2 mm in any direction (in cases of invasive lobular carcinoma or DCIS, at least 5 mm); and had no lymph or blood vessel invasion (L0, V0). For patients allocated to irradiation of the whole breast, two tangential opposing megavoltage (4–10 MV) photon beams were typically used. A total dose of 50.0–50.4 Gy was delivered with daily fractions of 1.8–2.0 Gy in 25–28 fractions. The tumor bed boost dose was 10 Gy in five fractions, delivered with electrons. For patients allocated to APBI, the clinical target volume consisted of the tumor bed with an adequate safety margin in all directions. The size of the safety margin (calculated as the sum of the width of the clear pathological surgical margin plus the radiation safety margin) had to be at least 20 mm, and this margin was defined individually for every patient. APBI was delivered with high-dose-rate (HDR) or pulsed-dose-rate (PDR) multicatheter brachytherapy. A total dose of 32 Gy in eight fractions (8 × 4.0 Gy) or 30.3 Gy in seven fractions (7 × 4.3 Gy), with fractionation twice a day, was used for HDR brachytherapy. A total dose of 50 Gy with pulses of 0.60–0.80 Gy/h (one pulse per h, 24 h/day) was given by PDR brachytherapy. Addressing non-inferiority, the analysis of this trial’s findings was not primarily based on the intention-to-treat principle, because this approach sometimes introduces bias towards no difference, which is anticonservative in this setting, i.e., would exaggerate estimates of equivalence. Instead, the primary analysis was performed “as treated”: after a median follow-up of 6.6 years, non-inferiority was reported for the 5‑year local control rate (1.4% APBI vs. 0.9% WBI, *p* = 0.42). Secondary (sensitivity) analyses, both a “per-protocol” analysis and an “intention-to-treat” analysis, were done to examine the consistency of results: 5‑year local recurrence was 0.97% or 1.07% (WBI) vs. 1.38% or 1.33% (APBI), respectively (*p* = 0.53 and *p* = 0.42). Disease-free survival (~94–95%) and overall survival (95–97%) were also similar in both arms. Moreover, a similar incidence of the majority of late side effects was shown. The cumulative incidence of any late side effect of grade 2 or worse at 5 years was similar in both groups, with 27.0% in the whole-breast irradiation group versus 23.3% in the APBI group (*p* = 0.12). However, the cumulative incidence of grade 2–3 late skin toxicity at 5 years was significantly different, with 10.7% in the whole-breast irradiation group versus 6.9% (4.8–9.0) in the APBI group (*p* = 0.020; [1, 3]). Finally, detailed analysis of quality of life questionnaires in this trial during follow-up showed that global health status was stable in both groups, but a moderate, statistically significant difference between the groups in the breast symptoms scale was found. Breast symptom scores were significantly higher, i.e., worse, after whole-breast irradiation than after APBI [4].

For single-catheter devices, the evidence is currently limited. In the NSBAP-B-39/RTOG 0413 trial, a subgroup of patients (23.3%) was treated with the MammoSite single-entry device. As described above, the first results of this trial were presented at the San Antonio Breast Cancer Symposium 2018; however, at the time of writing the presented guideline, corresponding subgroup analyses were not available. Thus, a valid assessment was not possible. For other brachytherapy APBI techniques, no phase 3 data are available.

### Intraoperative radiotherapy with electrons

In the ELIOT study [15], 1305 patients between 48 and 75 years of age and with tumors smaller than 2.5 cm were randomized to receive either single-dose intraoperative radiotherapy with electrons (IOERT) with 21 Gy (90% isodose) as PBI (experimental arm) or adjuvant WBI with 50 Gy in 25 fractions followed by an external electron boost of 10 Gy in 5 fractions (standard arm). Patients with four or more positive axillary nodes additionally received regional node irradiation up to 50 Gy (2 Gy/fraction). After a median follow-up of 5.8 years, significantly more in-breast recurrences were noted following full-dose IOERT (*n* = 35; 4.4%) than after WBI (*n* = 4; 0.4%; *p* < 0.0001). No significant difference in overall survival was observed and acute toxicity was lower in the experimental ELIOT arm. In a multivariate analysis for negative predictors, the highest risk for in-breast recurrence in the ELIOT arm was seen in patients with tumor sizes >2 cm, four or more positive lymph nodes, G3, and hormonal or triple-negative subtypes. Patients with at least one of these factors (*n* = 199) had a significantly higher risk of recurrence (11.5%, *p* < 0.0001) compared to those who had none (*n* = 452, 1.5%). These findings are corroborated by analyses of subgroups conducted among patients treated in several institutions [24–26]. In particular, the Milanese group investigated the outcome in 1822 out-trial patients treated solely by IOERT for low-risk breast cancer patients who were classified as “suitable” or “good” candidates according to the ESTRO/ASTRO guideline. Reported 5‑year recurrence risks amounted to 1.5% for 294 “ASTRO suitable” women and 1.9% for 573 “ESTRO good” candidates [24, 25]. Almost identical results were published by Maluta et al. from a phase II study in 226 low-risk breast cancer patients who received solely IOERT with 21 Gy: after a median follow-up of 62 months, 4 in-breast recurrences were noted, corresponding to a local relapse rate of 1.77% [26].

### Accelerated partial breast irradiation with 50-kV photons

For APBI with low-energy 50-kV photons, the Intrabeam device (Carl Zeiss Meditec, Oberkochen, Germany) delivers 50-kV photons to the tumor bed by using spherical applicators with a diameter of 15 to 50 mm that are inserted into the lumpectomy cavity. The prescribed dose is usually 20 Gy applied to the applicator surface. This dose attenuates to 5–7 Gy (depending on the applicator diameter) at a 1 cm distance from the applicator surface. The use of APBI with 50-kV photons with the Intrabeam system has been studied in the phase 3 TARGIT A-trial [14]. In this prospective randomized controlled phase III trial, patients were randomized to APBI with the Intrabeam system or whole-breast radiotherapy with or without a boost. Patients were eligible if they were ≥45 years old, had a tumor size ≤3.5 cm, and were candidates for breast-conserving surgery. Using a risk-adapted approach, patients in the experimental arm could receive additional whole-breast radiotherapy in case of further risk factors (lobular invasive cancer, extensive intraductal component). Results of 3451 patients were published with a median follow-up of 29 months [14]. The calculated 5‑year local recurrence rate was 1.3% in the standard arm and 3.3% in the experimental arm (*p* = 0.042, which was not considered statistically significant at the predefined significance level of 0.01). Using the absolute difference in the binomial proportions of local recurrence, non-inferiority was demonstrated. There was no statistically significant difference in terms of regional recurrences, breast cancer mortality, or overall survival; however, non-breast cancer-related mortality at 5 years was significantly lower in the experimental arm (1.4% vs. 3.5%; *p* = 0.0086). The authors separately assessed the risk of local recurrence in the pre-pathology (IORT given simultaneously during surgery) and post-pathology (IORT given after surgery as a second procedure by reopening the wound after the initial excision) strata. Non-inferiority could only be shown for the pre-pathology stratum (5-year local recurrence rate 2.1% vs. 1.1%), but not for the post-pathology stratum (5-year local recurrence rate 5.4% vs. 1.7%). To account for the relatively short median follow-up, the authors presented subgroup analyses for pre-pathology cohorts with a median follow-up 44 months (1450 patients) and 60 months (817 patients), yielding similar absolute differences in 5‑year local recurrence rates compared to the whole pre-pathology cohort. The significant difference regarding non-breast cancer-related mortality was restricted to the pre-pathology cohort. The incidence of grade III/IV radiotherapy-related skin complications was significantly lower in the experimental arm, although absolute numbers were small. A prespecified subgroup analysis according to the progesterone receptor (PR) status was published [27]. In the whole trial cohort, the 5‑year local recurrence rate for patients in the experimental arm compared to patients in the standard arm was similar in PR-positive patients (2.3% vs. 1.5%; *p* = 0.51), but higher for patients with PR-negative tumors (7.0% vs. 0.5%; *p* = 0.017). Corica et al. [28, 29] published results from a substudy of the TARGIT A-trial on quality of life and cosmesis. 126 patients treated in Australia were analyzed. The cosmetic outcome from different assessment methods favored the experimental arm, with significantly better cosmesis in the experimental arm at 5 years. Several quality of life subdomains also showed significant differences in favor of the experimental arm, among them the breast symptoms subdomain. Similar Abo-Madyan et al. [30] recently analyzed the long-term outcome of 184 patients enrolled onto the TARGIT A-trial at a single institution. Median follow-up was 8.5 years. There were only two local recurrences, resulting in a 5-year local recurrence rate of 0% (one recurrence after 70.3 months) for the experimental arm and 1.1% for the control arm (one recurrence after 4.5 months in a patient refusing all forms of adjuvant treatment). However, 42% and 14% of patients in the experimental arm received additional whole-breast radiotherapy or exclusive whole-breast radiotherapy, respectively. Thus, only 41% of patients in the experimental arm received IORT as the sole adjuvant radiotherapy treatment.

In total, the results of APBI with 50 kV IORT as presented in the TARGIT A-trial contain significant uncertainties and considerable limitations, as already heatedly discussed by many authors [31–38]. Criticism has centered on several aspects of the trial, which will be discussed in the following:

1. Duration of follow-up: The median follow-up of 29 months is immature and only 35% of the patients had 5‑year follow-up at the time of the analysis. This is especially important due to the high number of patients with hormone receptor-positive tumors, who have a risk of recurrence well beyond 5 years.
2. Non-inferiority design: The estimate used for the local recurrence rate of 6% at 5 years in the standard arm is considerably higher than what would be considered acceptable today. The TARGIT‑A authors used binomial proportions of local recurrence (i.e., number of recurrences divided by the number of patients) rather than Kaplan–Meier estimates of local recurrence rates. The use of binomial proportions has been criticized, since it does not take into account that only 1222 patients had a median follow-up of 5 years, which might lead to a dilution of the treatment effect. The authors presented results from cohorts with different follow-up times. Nevertheless, since these cohorts are nested within each other, the value of this analysis is questionable. Furthermore, as described in the NICE report 2018 [37], the TARGIT‑A investigators quantified the difference in the Kaplan–Meier estimates of local recurrence, and its 95% CI, using two different methods. The integrated difference method presented by the investigators is not commonly used, provided more favorable results for Intrabeam, and was not pre-specified in the TARGIT‑A protocol. Moreover, because the non-inferiority margin was based on the absolute difference in local recurrence, the same margin could not be used for assessing non-inferiority if the integrated difference method were to be accepted.
3. Use of whole-breast radiotherapy in the experimental arm: The trial used a risk-adapted approach for APBI. Thus, whole-breast radiotherapy could be added to APBI, which was the case for 15% of patients in the experimental arm. There were some pre-specified criteria for additional whole-breast radiotherapy, but each center could also add further criteria. There is no subgroup analysis of local recurrence rates in patients who received IORT alone. The use of additional whole-breast radiotherapy was considerably higher in the pre-pathology stratum (21.6%) than in the post-pathology stratum (3.6%), which might also have contributed to the better outcomes in the pre-pathology subgroup. This creates uncertainty as to whether IORT alone is a safe treatment option in certain patient subgroups.

In conclusion, in the face of these shortcomings, several international guidelines have discouraged the use of 50-kV IORT outside of clinical trials [39, 40]. This DEGRO expert panel concluded that because of the uncertainty of interpretation in the evidence available, the 50-kV system (Intrabeam) cannot be recommended for routine adjuvant treatment of early invasive breast cancer after breast-conserving surgery and should preferentially be used in the context of a clinical trial. Clinicians wishing to undertake APBI with 50-kV photons should ensure that patients understand the uncertainties about the procedure—particularly, patients should be counseled that follow-up is too short for general recommendations; that in corresponding clinical trial, still after very short not adequate follow-up, the risk of local recurrence was higher with APBI; and be informed about alternative treatment options [22, 37]. When used, it should be restricted to women with all of the following criteria: invasive cancer, aged >70 years, tumor <2 cm, resection margins >2 mm, grade 1— 2, pN0, ER positive, HER2 negative, L0, V0, and EIC negative.

## Recommendation

### General issues

We recommend that PBI with multicatheter brachytherapy or external beam radiation therapy after breast-conserving surgery (BCS) should be completed preferably in less than 12 weeks (typically within 6–10 weeks) and no later than 20 weeks, as better local tumor control and survival can probably be expected by keeping a shorter interval between BCS and radiotherapy [41–48]. Of note, a recent analysis illustrated that starting radiation therapy shortly after BCS seems not to be associated with a better long-term outcome [49]. If patients receive chemotherapy, PBI can be started after systemic treatment—in this scenario, we recommend starting PBI within 4 weeks after chemotherapy. It is also possible to start PBI before systemic treatment within 12 weeks. Radiation therapy can also be given in the interval between the chemotherapy courses.

In general, the physicians who indicate PBI have to reflect on the fact that level 1 evidence for non-inferiority of PBI in comparison to whole-breast irradiation is given for external beam techniques and for multicatheter brachytherapy [8, 50]. If the patient is interested in PBI with IORT techniques, the patient should be counseled in detail that there are uncertainties in the two available PBI phase 3 trials [14, 15]. One of the crucial problems of both PBI methods is that due to the lack of a final pathology report at the time of IORT, no definitive selection criteria could be applied at the time of PBI. As a consequence, the long-term results of PBI with electrons are only adequate if appropriately selected subgroups are analyzed [25, 26]. To warrant such appropriate selection at the time of breast-conserving surgery and IORT is very difficult. Some postulate that this patient selection is indeed prospectively possible, but no published data on this topic are currently available. Low-energy x‑ray IORT for PBI should be used within the context of a prospective registry or clinical trial; when used, it should be restricted to certain conditions, as discussed above.

### Selection criteria

Patient selection for PBI alone after BCS in patients with early breast cancer has been described in detail in European and US guidelines many times [22, 39, 51–55], and the credibility of different selection criteria has been proven in corresponding contemporary phase 3 trials [1, 2, 5, 6, 12–15]. Considering the fact only those APBI trials using strict selection are positive trials [1, 2, 12, 13] as well as corresponding deliberations in study protocols and current recommendations [22, 56], we recommend for daily routine, outside of any clinical trials, to only consider patients for PBI if all of following selection criteria are fulfilled:

1. Age ≥50 years.
2. Histology: any invasive carcinoma (any grade) or DCIS low to intermediate nuclear grade.
3. Tumor size: ≤3 cm (Tis, T1–T2).
4. Unifocal and unicentric DCIS or breast cancer.
5. Resection margins:
  - invasive cancer—negative by at least 2 mm,
  - invasive lobular histology or DCIS—negative by at least 5 mm.
6. No lymph vessel invasion (L0) and no hemangiosis (V0).
7. pN0/pNmi.

The following should be considered as contraindications for APBI:

1. Stage IIB–IV breast cancer.
2. Resection margins that cannot be microscopically assessed.
3. Extensive intraductal component (EIC).
4. Paget’s disease or pathological skin involvement.
5. Age ≤40 years.
6. Triple-negative or HER2-positive phenotype.
7. Neoadjuvant chemotherapy in treatment history.

Special attention should be paid to the practice of patient selection if APBI with intraoperative electrons or kV photons has been indicated. Here, the selection is a two-step process, consisting of a preoperative and an intraoperative phase. During the first-step selection, topographical, histological, and biological tumor features including radiological work-up are assessed. The second step takes place during surgery, based on histological examination of frozen sections, freedom of margins, and negative status of sentinel nodes. Nonetheless, it remains challenging to fully consider all APBI guidelines during delivery of IOERT when the final pathologic assessment is not yet available. In particular, the presence of lymphovascular invasion (LVI) and an extensive intraductal component (EIC) cannot be fully ruled out on preoperative core needle biopsy and intraoperative frozen section, leaving a margin of uncertainty for final eligibility. If definitive pathology is worse than anticipated, subsequent WBI might be necessary despite full-dose IORT.

### Target definition for APBI with multicatheter brachytherapy or EBRT

For target definition and delineation, irrespective of whether EBRT or brachytherapy techniques for PBI are intended, the ESTRO recommendations depending on the used BCS technique should be used [57, 58].

The following basic rules should be respected:

1. Detailed knowledge of the primary surgical procedure, of all details of the pathology report including size of resection margins in six directions, and of preoperative imaging (mammography and/or MRI and/or ultrasound) is obligatory.
2. Use of 4–6 surgical clips marking the borders of the lumpectomy is highly recommended for APBI.
3. Different resection margins in different directions should be respected. Total safety margins are defined as the sum of “size of existing surgical resection margin” plus “size of the added safety margin.” The recommended value of the total safety margin is 2 cm around the surgical bed considering the size of surgical resection margins in all six directions.
4. Target definition and delineation after closed-cavity surgery, oncoplastic cavity surgery, and after open-cavity surgery differ.

#### Target definition and delineation after closed-cavity surgery

**Fig. 1:**
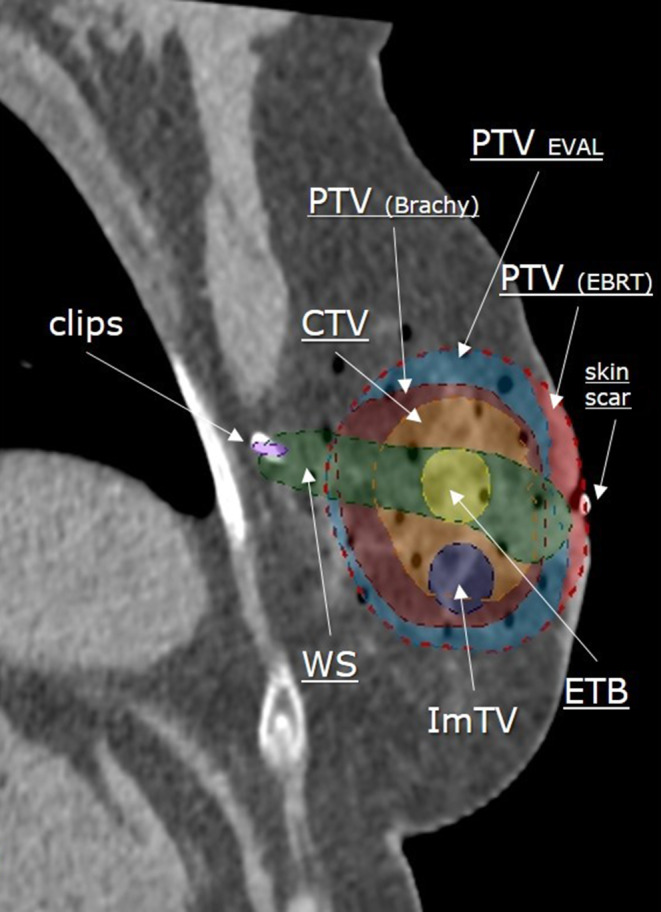
Target delineation after closed-cavity surgery. *WS* whole scar (*green*), *ImTV* imaging-correlated target volume (*dark blue*), *ETB* estimated tumor bed (*yellow*), *CTV* clinical target volume (*orange*), *PTV*_(Brachy)_ planning target volume for brachytherapy (*dark-red*), *PTV*_(EBRT)_ planning target volume for external beam radiation therapy (*light red*), *PTV*_EVAL_ planning target volume for evaluation (*light-blue*)

The target is related to the scar inside the breast and to the surgical clips. Use of CT imaging is standard. The following steps are proposed to delineate the target (see Fig. 1):

1. Delineation of the skin scar and of the clips.
2. Delineation of whole surgical scar (WS) inside the breast.
3. Delineation of ImTV (imaging-correlated target volume).
4. Delineation of ETB (estimated tumor bed).
5. Delineation of CTV (clinical target volume).
6. Delineation of PTV (planning target volume).

While delineation of the skin scar and the clips does not need further explanation, the delineation of next structures requires more attention. In summary, the “whole surgical scar” (WS) means delineating the whole visible surgical bed including the visible scar tissue inside the breast and all the clips—the whole “path” of surgeons inside the breast tissue, starting on skin scar and ending at the deepest point on the thoracic wall. Definition and delineation of ImTV (imaging-correlated target volume) can be done using only preoperative imaging and is based on the tumor size and tumor localization inside the breast. The next structure—estimated tumor bed (ETB)—is a reflection of three factors—“surgical clips,” “WS,” and “ImTV.” ETB is defined as the part of the whole scar which represented the tumor localization in the breast—strictly related to the localization and size of ImTV. The CTV is defined as ETB plus corresponding total safety margins—20 mm minus individual surgical margin in the corresponding direction, but at least 10 mm (for example: a surgical margin of 7 mm requires a safety margin of 13 mm). The thoracic wall and the skin are at no times a part of CTV. Definition of PTV is important if EBRT-based APBI is intended. In this case we recommend as standard to add an additional margin of ≥10 mm for the PTV, and the thoracic wall and skin can be parts of the PTV. Additionally, in such cases, a special feature—a “help structure” for meaningful analysis of DVH, termed PTV_EVAL_—should be also generated. PTV_EVAL_ corresponds to the PTV without thoracic wall, lung, skin, and air (Fig. 1). If multicatheter-based APBI is planned, then typically CTV = PTV, and only in the case of an absence of clips or impaired visibility of surgical scar or cavity do we recommend adding an adapted PTV margin (5–10 mm) in the region of doubt (excluding thoracic wall and skin). For more details, please see the corresponding guidelines [57].

#### Target definition and delineation after oncoplastic surgery

An important precondition here is that the position of surgical clips be intra-parenchymal and that placement occurs before the rotation of glandular tissue. Accordingly, the clipped area should be delineated in all corresponding CT slices as a surgical bed. As a consequence the “preliminary CTV” is defined, similar to the situation described above, as a “clipped area” plus corresponding total safety margins—meaning 20 mm minus the smallest individual surgical margin, but at least 10 mm (for example: a surgical margin of 2 mm requires a safety margin of 18 mm in all directions). If multicatheter brachytherapy is intended, the “final CTV” after oncoplastic surgery is defined as the “preliminary CTV” + 10 mm. If EBRT is intended, PTV is defined as the “preliminary CTV” + ≥20 mm. Concerning thoracic wall and skin, the same rules as those described above should be applied.

#### Target definition and delineation after open-cavity surgery

The target is related to the lumpectomy (seroma) cavity inside the breast and to the surgical clips, and use of CT imaging is standard. Lumpectomy cavity boundaries have to be defined by a combination of breast tissue changes apparent on CT images, in terms of tumor pathology and preoperative imaging, fluid collection within the lumpectomy cavity, and surgical clips when available. The CTV is defined as lumpectomy cavity plus corresponding total safety margins—20 mm minus individual surgical margin in the corresponding direction, but at least 5 mm (for example: a surgical margin of 6 mm requires a safety margin of 14 mm). The thoracic wall and the skin are at no times a part of CTV. For more details see please the corresponding guidelines [58].

### Target definition for APBI with IORT with electrons

IORT with electrons is performed after tumor excision and confirmation of negative margins, before oncoplastic reconstruction. In order to encompass sufficient breast tissue adjacent to the excised tumor, the walls of the excision hole are temporarily sutured to the center. The target volume has to encompass at least 20 mm in any direction with the exception of the skin, which is completely out of the irradiated volume, and the chest wall, which is usually protected by a shield disc. Tube diameters have to be chosen accordingly; diameters less than 5–6 cm are discouraged. The target volume thickness is assessed by intraoperative ultrasound or by inserting a needle probe at one or more representative points of the parenchyma, depending on the shape of the tumor bed and chest wall curvature. According to measurements, electron beam energies are selected to cover the depth with at least 90% of the prescribed dose (usually energies between 4 and 10–12 MeV). In case of tumors adjacent to the chest wall, the full depth distance to the shield is considered. Of note: in comparison to external techniques, no safety margins for set-up have to be considered.

### Target definition for APBI with IORT with low-energy photons (Intrabeam)

In principle, using IORT with the Intrabeam device, the target volume is predetermined by the surgical approach. Complete macroscopic excision of the tumor is required. Furthermore, based on histological examination of frozen sections, freedom of margins and negative status of sentinel nodes is required. Then, after insertion of the adequate spherical applicator (see above), the targeted volume corresponds to the volume adapted to the sphere and is determined by the size of the sphere.

## Techniques

### External beam radiation therapy

For external beam radiation-based APBI in supine and prone patient positions, different 3D external beam techniques with non-coplanar or with mini-tangent beams in combination with an en face electron field, step and shoot (SS) and sliding window (SW) intensity-modulated radiotherapy (IMRT) techniques, and VMAT/intensity-modulated arc therapy techniques have been used in several prospective trials [2, 12, 13, 59–69]. In principle, independent of the EBRT technique, the same principles of EBRT as for other indications are applied. Typically, the 3D-CRT plans are created with four or five wedged conformal non-coplanar fields from tangential directions, the IMRT plans with five or six coplanar fields, and the intensity-modulated arc therapy plans consist of two or more coplanar arcs. Adequate target volume coverage and acceptable doses to the organs at risk are achievable with all techniques. Some analyses recommend the SW-IMRT technique as the best EBRT technique for APBI [70]. The reproducibility and precision of the position of the patient and of the tumor should be verified with available imaging tools. When, as strictly recommend, surgical clips are in situ, 2D imaging is often sufficient to achieve appropriate positioning and the kV-kV planar imaging method is recommended. Without surgical clips in situ, 3D images should be acquired, and the recommended method of verification is the kV cone beam CT [71]. Image-based verification of reproducibility and precision should be performed before each fraction of APBI.

The prescribed dose should be specified respecting ICRU 50 and ICRU 62 at reference point (typically corresponding with isocenter), and dose–volume histogram analysis of target coverage should confirm that 100% of the prescribed dose covers >90% of the PTV/PTV-EVAL. Restrictions for surrounding tissues should be respected. Using EBRT for APBI, the dose constraints for the target and for organs at risk are identical to those described below for brachytherapy and are summarized below.

### Brachytherapy

Brachytherapy techniques represent the most investigated irradiation techniques for APBI. Regarding the performance, features, and reproducibility as well as regarding the possibilities to shape adequate dose distribution, there exist large differences among diverse brachytherapy techniques. Today there are several brachytherapy techniques available—multicatheter brachytherapy; single-entry intracavitary devices like the MammoSite balloon; single-entry multilumen catheter devices like SAVI (strut-adjusted volume implant, Cianna Medical, Aliso Viejo, CA), CONTURA Multi-Lumen Balloon (Bard Biopsy Systems, Tempe, AZ), or ClearPath (NorthAmerican Scientific Inc., Chatsworth, CA); electronic brachytherapy, seeds brachytherapy, and non-invasive brachytherapy [72–81]. Among all these techniques, level 1 evidence is only available for multicatheter brachytherapy [1].

For image-guided multicatheter brachytherapy-based APBI, we recommend in general to respect basic rules of image-guided interstitial brachytherapy for breast cancer [82, 83]. Treatment planning and catheter insertion depend in detail on the availability of appropriate imaging facilities (x-ray, CT, MRI, ultrasound) and on the kind of breast-conserving surgery (open cavity, closed cavity, plastic reconstruction). Treatment planning and catheter insertion should be performed according current ESTRO-ACROP guidelines [84]. Furthermore, the catheter reconstruction, normalization of dose distribution, dose specification, dose prescription, optimization methods, and quality management issues should be implemented in line with this guideline [84].

### IORT with electrons

Technical details of IOERT have been published repeatedly [85, 86]. IOERT is nowadays mostly performed on mobile linear accelerators with tube sizes of 5–8 cm in diameter and electron energies usually ranging from 4–12 MeV according to a given target volume (see above).

### IORT with 50-kV photons

The Intrabeam device provides a point source of 50-kV x‑rays at the center of a spherical applicator. Applicator diameters ranging from 1.5–5.0 cm are available.

After tumor removal, the size of the sphere is determined by the radio-oncologist in close collaboration with the breast surgeon. The spherical applicator is then inserted into the surgical cavity. The appropriately sized applicator should fit comfortably without tension in the surrounding tissue. Subsequently, the subcutaneous tissues will be gathered with a purse-string suture over the sphere to adapt the target breast tissue well to the surface of the applicator sphere. Also, at the bottom of the resection cavity, the breast tissue should be adapted to the applicator surface, i.e., contracting the tissue using a purse-string suture, if necessary.

The skin, but not the breast tissue, should be kept away from the applicator using, e.g., a piece of wet gauze to prevent direct contact. It is suggested to keep the skin at a distance of at least 1 cm from the sphere. For tumors near to the skin (≤1 cm), an elliptical piece of overlying skin should be excised.

The calculation of the radiation dose to the heart, left ventricle, and especially to the left anterior descending branch of the coronary artery the thickness of the chest wall (muscle and rib cage) should be always perfomed. Possibly, if there is no adequate distance, the surface of the applicator sphere should be kept away or covered with a protective cap at the chest wall. However, in most patients, the normal thickness of the chest wall (muscle and rib cage) may provide adequate shielding.

## Dose–volume parameters and dose constraints

For an objective assessment of any treatment plan, quantitative parameters have to be analyzed and reflected. It’s in the nature of things that such appropriate objective analysis is not possible for PBI using IORT techniques. Table 2 lists the most common dose–volume parameters used in PBI with interstitial multicatheter breast brachytherapy and EBRT.

**Table 2:** The most common dose–volume parameters used for reporting partial-breast irradiation. (Adapted according [84])

Parameter	Definition/calculation
*Reference dose related*	
V_PD_	Absolute volume irradiated by the prescribed dose
V_1.5xPD_	Absolute volume irradiated by 1.5 x the prescribed dose
DNR—dose non-uniformity ratio* (only for brachytherapy)*	V1.5xPD/VPD
DHI—dose homogeneity index* (only for brachytherapy)*	(VPD − V1.5xPD)/VPD
*Target related*	
V_PTV_	Volume of the PTV
V_xx_	Percentage of PTV receiving xx% of the PD
CI—coverage index (V_100_)	V100/100
COIN—conformal index	PTVPD/VPTV PTVPD/VPD
D_xx_	Percentage dose that covers xx% of the PTV
*OAR related*	
Dmean	Mean dose in organ
V_xGy_	Relative volume x receiving Gy
V_xx_	Percentage of organ receiving xx% of the PD
D_xcm3_	Relative dose given to most exposed x cm3 of organ

*PD* prescribed dose, *PTVPD* volume in planning target volume receiving at least the PD

Based on the ESTRO-ACROP guideline [84] and NSABP Protocol B-39/RTOG 0413 [87], we recommend the following target-related dose–volume limits:

1. Coverage index (CI): V_100_ ≥ 90–95% (i.e., at least 90% of the PTV had to receive the PD)
2. V_150_ <65 cm^3^
3. V_200_ <15 cm^3^
4. Absolute volume irradiated by prescription dose—D_PD_ ≤300 cm^3^
5. Dose non-uniformity ratio—DNR ≤35 (only for brachytherapy)
6. Conformal index—COIN ≥65 (only for brachytherapy)
7. Dose homogeneity—maximal dose should not exceed 110% of prescribed dose (only for EBRT)

The recommended dose–volume limits for OARs [10, 84, 87], according the current available data, are presented in Table 3.

**Table 3:** Recommended dose–volume limits for organ at risk. (Modified according [84])

Organ	Constraints
Ipsilateral non-target breast	V_90_ <10% V_50_ <40(50)%
Skin	D_1cm3_ <90% D_0.2cm3_ <100%
Rib	D_0_._1cm3_ <90% D_1cm3_ <80%
Heart	Mean heart dose, MHD <8% (<2.5 Gy) D_0.1cm3_ <50%
Lung	Mean lung dose, MLD <8% (<3–4 Gy) D_0.1cm3_ <60% stochastic effects: MLD <1–1.5 Gy

*MHD* mean heart dose, *MLD* mean lung dose, *V* volume, *D* dose

For IOERT, the following dose reports and constraints are recommended: the dose is prescribed at D_max_ (100%) or at D_90_. D_max_, D_90_, D_45_ and their corresponding tissue depths (d) should be specified along the central beam and clinical axis (in mm), respectively. V90 is defined as the tissue volume which is encompassed by the 90% isodose and should be indicated in ml (cc). V_90_ can be calculated by the formula of a rotating ellipsoid (4 × 3.14/3 × a2 × b).

During IOERT, the skin is outside the treated area and thus not affected by radiation. In rare cases when IOERT takes place without a chest wall protection shield, the dose to the anterior rib surface should not exceed 10 Gy. In case of shield use, the rib dose falls by nature far under this value. Likewise, exit doses to lung and—in case of left-sided BC—heart structures amount to less than 1 Gy, which is not considered as clinically relevant.

## Dose schedule

### External beam radiation therapy

The recommended schedules with EBRT are:

1. Total dose 40 Gy, 2.66 Gy in 1 fraction/day ~15 fraction over 3 weeks
2. Total dose 38.5 Gy, 3.85 Gy in 1 fraction/day ~10 fraction over 10 days

### Brachytherapy

The recommended schedule with PDR brachytherapy is total dose 50 Gy, pulsed-dose 0.5–0.8 Gy/pulse, scheduled every hour, 24 h per day, total treatment time 4–5 days.

The recommended schedules for HDR brachytherapy are 8 × 4 Gy, 10 × 3.4 Gy, and 7 × 4.3 Gy, twice per day, with an interval between fractions of at least 6 h, and with a total treatment time of 4–5 days.

Other fractionations can be used. However, the chosen fractionation should correspond to a biologically equivalent total dose EQD2 (a/b = 4–5 Gy) in the range of 42–45 Gy.

### IORT with electrons

Full-dose IOERT is to date most frequently performed with 21 Gy, prescribed at the depth of the 90% isodose line (Dmax 23.3 Gy).

### IORT with 50-kV photons

The prescribed dose is usually 20 Gy applied to the applicator surface. This dose attenuates to 5–7 Gy (depending on the applicator diameter) at a distance of 1 cm from the applicator surface. Thus, the tumor bed typically receives 20 Gy, at the surface this attenuates to 5–7 Gy at a depth of 1 cm.

The dose to the skin should be <7 Gy. This can be realized by keeping the skin ≥1 cm away from the sphere.

## Conclusion

APBI has been tested in a total of nine phase 3 trials with more than 15,000 patients over the past 10 years [1, 2, 5, 6, 12–16]. These trials show that for strictly selected patients with early breast cancer, PBI by EBRT, multicatheter brachytherapy, or IORT with electrons is non-inferior to the results of whole-breast irradiation in terms of local control, disease-free survival, and overall survival, and is in some aspects superior regarding late side effects and quality of life.

Furthermore, we conclude that PBI requires expertise encompassing:

1. appropriate patient selection.
2. appropriate target delineation considering the applied technique of breast-conserving surgery.
3. a high-level quality assurance program regarding the respective technique of PBI.

In light of current data, PBI using multicatheter brachytherapy, EBRT, or IORT with electrons is a valid alternative treatment option after breast-conserving surgery and can be offered for carefully selected low-risk breast cancer patients in clinical routine using the proposed selection criteria.
